# Role of *Tafazzin* in Mitochondrial Function, Development and Disease

**DOI:** 10.3390/jdb8020010

**Published:** 2020-05-23

**Authors:** Michael T. Chin, Simon J. Conway

**Affiliations:** 1 Molecular Cardiology Research Institute, Tufts Medical Center, Boston, MA 02111, USA; 2 Herman B. Wells Center for Pediatric Research, Indiana University School of Medicine, Indianapolis, IN 46202, USA

**Keywords:** *Tafazzin*, mitochondria, Barth syndrome, rare X-linked genetic disease, cardiolipin, heart failure, left ventricular noncompaction

## Abstract

*Tafazzin*, an enzyme associated with the rare inherited x-linked disorder Barth Syndrome, is a nuclear encoded mitochondrial transacylase that is highly conserved across multiple species and plays an important role in mitochondrial function. Numerous studies have elucidated the mechanisms by which *Tafazzin* affects mitochondrial function, but its effects on development and susceptibility to adult disease are incompletely understood. The purpose of this review is to highlight previous functional studies across a variety of model organisms, introduce recent studies that show an important role in development, and also to provide an update on the role of *Tafazzin* in human disease. The profound effects of *Tafazzin* on cardiac development and adult cardiac homeostasis will be emphasized. These studies underscore the importance of mitochondrial function in cardiac development and disease, and also introduce the concept of *Tafazzin* as a potential therapeutic modality.

## 1. Introduction

Mitochondria are ubiquitous essential cellular organelles that control mitochondrial respiration and adenosine triphosphate (ATP) generation through the metabolism of key substrates, thereby regulating cellular energy balance. Mitochondria are also unique among organelles in that they contain their own genome and translational apparatus that allows synthesis of a small set of essential proteins specific to the mitochondria. The mitochondrial proteome, however, is complex and includes many proteins that are encoded in the nuclear genome, synthesized in the cytoplasm and transported across the mitochondrial outer membrane, where they are sorted and transported to either the outer membrane, intermembrane space, inner membrane or mitochondrial matrix. Mitochondrial dysfunction is a common feature of many medical disorders, while primary mitochondrial disorders invariably affect multiple organ systems simultaneously. The role of mitochondria in development is poorly understood. This review will discuss the role of *Tafazzin* (*Taz*) in mitochondrial function, organism development (which is often overlooked and underappreciated) and within human disease by highlighting important findings and identifying unmet needs.

## 2. *Tafazzin* as the Genetic Cause of Barth Syndrome

Barth Syndrome (BTHS) was first described as an X-linked syndrome of dilated cardiomyopathy, neutropenia and skeletal myopathy resulting in death in male infancy or early childhood from septicemia and/or cardiac decompensation [1]. Mitochondrial respiratory abnormalities were noted in cultured fibroblasts [2]. The gene associated with BTHS, originally designated G4.5, was identified in 1996 [3] and renamed *Tafazzin*, after a popular Italian television character, Mr. Tafazzi, who was known for his comedic self-flagellation. The name change was proposed due to early difficulties in characterizing the function of the gene. The human gene, located on Xq28, consists of 11 exons and was originally reported to encode multiple alternatively spliced mRNAs differing through the use of two different 5′, tissue-specific promoters and through selective splicing of exons 5, 6 and 7, theoretically resulting in up to 10 distinct mRNAs [3], with the most abundant species consisting of the full-length transcript and one lacking exon 5, which encodes a hydrophilic domain of unknown function and significance. Later studies were only able to confirm the presence of four distinct mRNAs in human samples [4,5], consisting of a full-length transcript, a transcript lacking exon 5, a transcript lacking exon 7 and a transcript lacking both exons 5 and 7. However, the possible role/s of these various isoforms have yet to be functionally investigated. Intriguingly, this gene is highly conserved across multiple species, including yeast, nematodes, zebrafish, frogs and fruit flies, but exon 5 is primate specific and encodes an unstructured, hydrophilic domain [5,6,7]. Table 1 highlights the major model organisms used to examine and understand *Tafazzin* requirement, organization and function. It lists the species and their associated major phenotypes, as well as the type of genetic manipulation and whether it results in absent *Tafazzin*, reduced wildtype *Tafazzin* or presence of mutant *Tafazzin* protein.

## 3. Tafazzin Is a Conserved, Genomically Encoded Mitochondrial Transacylase That Is Transported into the Mitochondria and Remodels Cardiolipin

**Figure 1:**
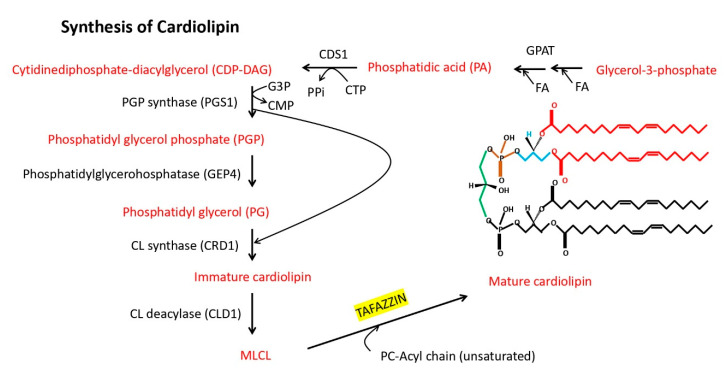
Biosynthesis of yeast cardiolipin (CL) within inner mitochondrial membrane. The initial steps of cardiolipin synthesis involve fatty acid esterification of glycerol-3-phosphate by glycerol-3-phosphate O-acyltransferase (GPAT) to generate phosphatidic acid (PA), which subsequently is modified by phosphatidate cytidylyltransferase 1 (CDS1) to form CDP-diacylglycerol (CDP-DAG). CDP-DAG then is modified by phosphatidylglycerolphosphate synthase (PGS1) to form phosphatidylglycerol phosphate (PGP), which is then modified by phosphatidylglycerophosphatase (GEP4) to phosphatidyl glycerol (PG). PG is then modified by cardiolipin synthase (CRD1) to form immature cardiolipin, which is then processed to MLCL by cardiolipin-specific deacylase 1 (CLD1). The yeast *Tafazzin* ortholog then reacylates MLCL to mature CL in mitochondria using a phosphatidylcholine acyl chain donor.

Early homology studies based on the amino acid sequence suggested a relationship to acyltranferases [6]. Initial clinical studies on cells derived from BTHS patients indicated a defect in remodeling of monolysocardiolipin to mature cardiolipin and an overall decrease in mature cardiolipin content [20,21,22,23,24], suggesting that the primary enzymatic function of *Tafazzin* is to generate mature cardiolipin within the mitochondria (see Figure 1). Cardiac and skeletal muscle have high levels of cardiolipin and, therefore, are most affected in BTHS [25]. Studies in yeast lacking activity of the *Tafazzin* orthologue Taz1p showed a similar defect in cardiolipin remodeling that was rescued by a human *Tafazzin* cDNA lacking exon 5 [8,26]. Direct measurement of the enzymatic activity of recombinant *Drosophila Tafazzin* confirmed the ability of the enzyme to remodel cardiolipin [27]. Follow up studies have differed on whether the enzyme is substrate specific [28,29]. The most recent study indicates that the enzyme recognizes structure rather than fatty acid side chain and thus can remodel monolysocardiolipin to various forms of mature cardiolipin, and suggests that earlier discrepancies resulted from the use of nonionic detergent in the assays [30]. Nevertheless, the remodeling of cardiolipin is hypothesized to ease strain during the packing of lipids that arise during formation of membrane curvature [31]. Cardiolipin is a key component of the inner mitochondrial membrane, where it represents about 20% of the total lipid composition and is essential for the optimal functioning of numerous enzymes involved in mitochondrial energy metabolism. Moreover, cardiolipin is the only phospholipid specific to mitochondria and is vital for normal mitochondrial structure and function including electron transport chain assembly to synthesize ATP [22,32]. Pathological changes in cardiolipin amounts and/or species composition can have harmful consequences for mitochondrial function and trigger the production of reactive oxygen species (ROS). Specifically, accumulation of intermediate monolysocardiolipin (MLCL), a key diagnostic marker of BTHS [25], itself may contribute to mitochondrial dysfunction via cytochrome C release and/or activation of the proapoptotic pathway [33]. Additionally, cardiolipin can act as a signaling platform, as when it is exposed to the outer mitochondrial membrane upon mitochondrial stress, the cardiolipin domains can serve as a binding site in many cellular signaling events [32].

Numerous studies have been performed to elucidate the relationship between *Tafazzin* structure and function. Analysis of enzymatic activity of the four human cDNA-encoded species and three *Drosophila*-encoded species demonstrated that the full length cDNA and the cDNA lacking exon 5 demonstrated enzymatic activity and ability to rescue the *Drosophila* mutant phenotype, although the full length protein was less strongly integrated into the hydrophobic core of the inner mitochondrial membrane than the protein lacking exon 5 [34]. At present, the functions of the ∆7and the ∆5∆7isoforms have not been determined.

Although the crystal structure of *Tafazzin* has not yet been determined, structural prediction algorithms have been used to infer domain structure [6,7]. A 21 amino acid transmembrane domain is predicted in the N-terminal region encoded by exon 1, a putative catalytic HX4D motif is identified in exon 2 and a putative 57 amino acid substrate binding cleft spanning exons 2, 4, 6, 7, 8 and 10 is also present. These findings are consistent with published work showing that isoforms lacking exon 7 are not enzymatically active [34].

Examination of naturally occurring variants in the BTHS patient population reveals a broad spectrum of mutations that occur in all exons, including exon 5, indicating that there is no “hot spot” for disease causing variants [5,35] (https://www.barthsyndrome.org/research/Tafazzindatabase.html). Functional analysis of the various human mutations has been performed mostly through a series of experiments where disease associated variants have been introduced into the Taz1p yeast orthologue with subsequent investigation into the effects on yeast protein function [36,37,38,39]. An initial study demonstrated that Taz1p associates with the inner mitochondrial membrane through a membrane anchor domain within residues 215–232 and that introduction of the missense variants V223D, V224R, I226P and G230R altered the ability of the protein to rescue the ∆Taz1p phenotype, characterized by growth retardation and accumulation of MLCL. V223D, V224R and I226P resulted in mis localization to the mitochondrial matrix, while G230R led to altered protein complex formation [36]. Analysis of the A88R/E, S40R and L148H variants identified an additional potential disease mechanism whereby mutated Taz1p proteins underwent degradation by the i-AAA protease, most likely due to protein instability and aggregation [37]. Further characterization of additional missense mutations led to the identification of other functional classes: catalytically inactive, reduced catalytic activity and thermosensitivity [39]. The functional classification of some of these variants was later documented in human *Tafazzin* within human cells [38]. However, the significance of the primate-specific *Tafazzin* exon 5 has yet to be functionally determined [5,6,7].

The mechanism by which *Tafazzin* is imported into mitochondria and transported to the interfacial surfaces of the outer and inner mitochondrial membrane has also been studied in yeast. Taz1p is imported by the outer membrane translocase complex and then uses the Tim9p-Tim10p complex of the intermembrane space to insert into the outer membrane. From there it is sorted to the inner membrane via intermediate density membranes. Overall localization was dependent upon the membrane anchor domain at amino acid residues 215–232 [40]. A study of human TAZ peptides fused to green fluorescent protein in mammalian cells identified a similar mitochondrial membrane anchor domain located within amino acids 185–220 and a novel mitochondrial localization domain located within amino acids 80–95 [41]. Whether BTHS phenotype/s in vivo may be caused by altered *Tafazzin* spaciotemporal localization is unclear, although these in vitro studies are suggestive of a role of correct cellular targeting as a potential pathogenic mechanism.

## 4. *Tafazzin* Is a Regulator of Mitochondrial Structure and Function

Ultrastructural abnormalities in mitochondria were noted in the original case report describing BTHS [1], and have been associated with defective mitochondrial Complex III function [2], but the nature of BTHS-mediated mitochondrial dysfunction has been complex and variable. In yeast, early reports indicated a defect in energy coupling and membrane stability [8], while studies in patient-derived lymphoblasts indicated abnormal proliferation, altered membrane potential and normal ATP formation, suggesting partial uncoupling and compensatory expansion of the mitochondrial compartment [42]. Changes in ultrastructure are also variable dependent upon model system and organ analyzed, and may be more prominent in differentiated rather than embryonic tissues. The effect has been hypothesized to be greater in mitochondria with higher cristae stacking density [43] and as heart mitochondria have twice the diameter and higher percentages of lamellated cristae (cristae increase surface area and allow for inner membrane cardiolipin assembly) than other organs, this may help explain why BTHS patients exhibit predominantly cardiovascular defects [44] Abnormal cardiolipin remodeling due to *Tafazzin* deficiency leads to destabilization of mitochondrial inner membrane complexes in yeast [45], disrupts respiratory super complex formation in patient lymphoblasts [46] and also interferes with super complex formation in human iPS cells [47]. Interestingly, despite alterations in cardiolipin profiles and disruption in mitochondrial respiratory super complexes, metabolic flux through the TCA cycle was not disrupted in patient skin fibroblasts [48]. In Drosophila flight muscles, the density of the F_1_F_0_ ATP synthase dimers in the inner mitochondrial membrane was reduced at high curvature areas and dimer rows were less extended and more scattered [49]. In mice containing a doxycycline inducible shRNA that knocks down *Tafazzin* expression, mitochondria developed a wide spectrum of mitochondrial abnormalities [12,13,14]. Both the morphological defects (i.e., mixture of swollen, honeycomb and widened/collapsed/absent cristae) and numbers of mitochondria are variable, as there are reports of increased, decreased and unchanged mitochondrial numbers, depending upon which stage, which organs and doxycycline concentration used. An extensive bioenergetic and lipidomic characterization of these mice revealed differential substrate utilization, and reduction in Complex III and V activities [50]. An independent assessment of mitochondrial function in the same line of mice and in human iPS cell-derived cardiomyocytes (iPSC-CMs) from BTHS patients indicated a tissue-specific reduction in Complex II succinate dehydrogenase activity [51]. A separate BTHS iPSC-CM study implicated a reduction in F_1_F_0_ ATP synthase specific activity and overall ATP reduction in cells cultured in galactose, to limit ATP generation from glycolysis [19]. Interestingly, basal oxygen consumption rate is increased in BTHS iPSC-CMs, likely due to compensatory mechanisms, but total respiratory capacity is decreased [19,51]. *Tafazzin*-deficient mitochondria have been noted to generate increased ROS in yeast [52], in *Tafazzin* knockdown mice [51] and in human iPSC-CMs [19].

## 5. The Role of *Tafazzin* in Cellular Differentiation and Development

*In vitro* studies on cultured cells have suggested a role for *Tafazzin* in regulation of differentiation. BTHS iPSC-derived cardiomyocytes show irregular sarcomere organization but no difference in differentiation efficiency when cultured for 60 days [51] or when cultured on an un-patterned substrate [19]. This defect in sarcomere organization was only seen with a *Tafazzin* frameshift variant (c.517delG) but not a missense variant (c.328T > C), however, engineered tissue constructs generated from iPSC-CMs containing each variant demonstrated contractile dysfunction [19]. Targeted disruption of the endogenous *Tafazzin* locus in a C2C12 mouse myoblast cell line led to altered differentiation into myotubes [11].

The effect of *Tafazzin* loss of function on *in vivo* organismal development has been studied in a variety of model organisms. In zebrafish embryos, at 10 h post fertilization (hpf), *Tafazzin* is expressed ubiquitously with strongest expression in the head area, at 24 hpf is expressed highly in the head, eye and tail and by 30 hpf, becomes more restricted to the head, heart, eyes and region next to the yolk corresponding to endodermal tissue. At 51 hpf, *Tafazzin* mRNA becomes highly restricted to the zebrafish heart, although this intriguing tissue specificity has yet to be confirmed in mammals or in later mature hearts. Morpholino antisense oligonucleotide-directed knockdown of *Tafazzin* mRNA led to severe developmental abnormalities in a dose-dependent fashion. The most severely affected structures were in the heart and tail, with some eye abnormalities observed. Morphant embryos at 51 hpf developed marked edema with large pericardial effusions associated with dysmorphic, slowly beating hearts. The heart tube failed to loop, showing a profound effect on heart development. Co-injection of zebrafish *Tafazzin* mRNA containing a variant analogous to the human mutation G197R rescued bradycardia and tail abnormalities but continue to demonstrate heart failure [10].

In Drosophila, generation of *Tafazzin* null mutants by imprecise P-element excision upstream of the start codon in exon 1 was associated with abnormal cardiolipin remodeling and abnormal mitochondrial morphology as described above. Lifespan was unchanged, and heart rate was unchanged in pupae and locomotor activity was normal in larvae. Quantitative measurement of motor weakness in adult flies demonstrated reduced ability to climb against gravity [9]. The relatively mild effect of *Tafazzin* deletion on organism development in Drosophila is likely a reflection of the significant differences between the circulatory systems of insects and vertebrates.

In mice, an understanding of the role of *Tafazzin*, cardiolipin and mitochondria in organism development are each incompletely understood. For instance, it is unknown when *Tafazzin* mRNA or protein is initially present within mouse embryos, if there is differential expression levels and when mature cardiolipin is first detectable and within which organs. Similarly, although it is known that mouse embryonic heartbeat starts at five somites (around embryonic day E8) and that blood flow is initiated at seven somites (around embryonic day E8.25), it is unknown when and where the first mitochondria are developed [53,54]. Moreover, as the embryonic heart is thought to initially rely solely on anaerobic glycolysis prior to placental development establishing circulation and rising oxygen levels, it is thought that mitochondria (and hence *Tafazzin* function) are presumed to be required as the in utero heart transitions to using subsequent aerobic respiration and an increasing reliance upon mitochondrially generated ATP [55,56,57,58]. Until recently, generation of fertile chimeras containing knockout alleles have been unsuccessful, most likely due to profound effects on spermatogenesis [15]. To date, most of our understanding of the role of *Tafazzin* in mouse development has been obtained from a doxycycline induced shRNA knockdown model of *Tafazzin* gene expression [12,13,14]. Knockdown induced via doxycyline from the start of gestation resulted in prenatal loss from E12.5–13.5, associated with myocardial thinning, hypertrabeculation, noncompaction and defective ventricular septation. Diastolic dysfunction was also noted. The effect on cardiovascular morphogenesis was recapitulated when knockdown was induced at E7.5 and at E10.5 but not at E13.5 or E14.5, suggesting an important developmental window for *Tafazzin* function. *Tafazzin* knockdown was associated with reduced compact zone proliferation but no apoptosis. Some embryos survived gestation, but none survived to adulthood [14]. Mitochondrial and cardiolipin abnormalities were as described above. This report differed from the other mouse studies by administering doxycycline at a higher dose in the drinking water compared to the other studies that used doxycycline containing chow [12,13], which may explain why the same strain of mice receiving doxycycline in chow showed predominantly an adult phenotype. Doxycycline, however, is known to have inhibitory effects on mitochondrial morphology/function itself [59] and continual administration of doxycycline to mice has been shown to adversely affect both cardiac and neutrophil function [60]. Thus, it is challenging to discern from this model whether the effects observed are as a direct result of the reduction of *Tafazzin* function, or a consequence of doxycycline exposure on the mitochondria, or even a combination of both. Another limitation of this model is that it relies on reduced expression of a normal *Tafazzin* gene rather than expression of pathogenic *Tafazzin* variants seen in patients. At present it is not clear whether loss of *Tafazzin* globally or specifically in the developing heart (or specific heart lineages) is responsible for the developmental and adult cardiac phenotypes observed. A recently developed *Tafazzin* conditional allele mouse line has enabled the study of both tissue specific and global deletion of *Tafazzin* [16]. Systemic deletion of *Tafazzin* in mice resulted in significant embryonic and perinatal lethality, while late embryonic deletion specifically in the heart resulted in adult onset dilated cardiomyopathy without any evidence of cardiac myocyte cell hypertrophy [17]. Systemic deletion also revealed that male *Tafazzin* knockout mice are sterile, as global loss inhibited germ cell meiosis as demonstrated by the reduced abundance of round spermatids and the near absence of elongated spermatids [16]. Although BTHS is thought to preferentially affect males, this result was unexpected and highlights the usefulness of transgenic mice approaches, as male infertility was not usually associated with BTHS. Further analysis of the embryonic phenotype or the tissue-specific contribution of *Tafazzin* to embryonic development using hematopoietic, testis, skeletal or early cardiac organ-restricted and lineage-specific deletion strategies has not yet been done, but is more than likely to be highly informative.

## 6. The Role of *Tafazzin* in Barth Syndrome, Non-Inherited Diseases and Potential Therapies in Development

The myriad of clinical features of BTHS have been well described [61,62]. The contribution of *Tafazzin* to mitochondrial dysfunction and subsequent muscle differentiation and dysfunction is easily understood given the enrichment of mitochondria in striated muscle tissue and the heightened energetic demands of muscle contraction. The contribution to left ventricular noncompaction can also be understood in this context as well, as the thickened left ventricle is the predominant chamber as it is responsible for pumping oxygenated blood throughout the entire body. The clinical impact of *Tafazzin* mutation on fetal loss and stillbirth has also been documented [63], although a clear effect on cardiovascular morphogenesis aside from noncompaction has not been described. Although the presence of cyclic neutropenia is also well documented in some BTHS patients, the mechanism causing this phenotype is poorly understood. Neutropenia makes it more difficult for the body to fight off foreign invaders such as bacteria and viruses, so affected individuals have an increased risk of recurrent infections. There is some evidence that apoptosis of myeloid precursors in the bone marrow results from *Tafazzin*-deficiency [64], but an explanation of why these cells are particularly susceptible has not been presented. Similarly, it is unclear why the high energy-requiring central nervous system is spared in BTHS, but not in many other mitochondrial diseases [65]. Table 2 lists the most common male BTHS patient phenotypes and their possible in utero and perinatal developmental origins. Although female carriers of *TAZ* mutations are usually asymptomatic and typically undergo selective inactivation of X chromosomes carrying the mutant allele, a couple of reports have shown that affected *TAZ* female carriers with both abnormal and normal karyotypes can exist [66,67,68]. This presumably occurs due to random X inactivation in utero leading to a high percentage of cells silencing the wildtype allele [69].

Reduction in mature and total cardiolipin has also been described in a spontaneously hypertensive rat model of heart failure, as well as adult human myocardial samples from patients with idiopathic dilated cardiomyopathy (IDCM) [70]. A subsequent follow up study found that the *Tafazzin* mRNA is reduced in both the hypertensive rat hearts and IDCM patient hearts but not control samples [71]. An analysis of pediatric cardiomyopathy samples demonstrated a similar reduction in mature and total cardiolpin in failing pediatric hearts but normal mitochondrial content and no change in *Tafazzin* expression, suggesting that other regulators of cardiolipin metabolism such as MLCL-AT may be important in the development of heart failure [72]. Increased *Tafazzin* expression has been associated with tumorigenicity of cervical cancer cells [73] and also with tumorigenesis and radiation response in rectal cancer [74]. Moreover, recent data has shown that *Tafazzin* can reduce stemness and increase differentiation of acute myeloid leukemia cells [75], although whether these are all primary or secondary effects upon cancer stem cells remains unknown.

Published therapeutic interventions for BTHS have focused on nutritional supplementation, reduction of oxidative stress and gene replacement therapy. The predominant mature cardiolipin in human hearts is tetralinoleoyl cardiolipin (L4CL) and BTHS patients show significant reduction in L4CL. Linoleic acid treatment of patient skin fibroblasts restores cardiolipin levels [18] and also partially improves BTHS iPSC-CM performance [19]. Scavenging of ROS in BTHS iPSC-CMs with mitoTEMPO also improved sarcomere organization and contractility [19], but scavenging of ROS *in vivo* through the use of mitochondrially targeted catalase in *Tafazzin*-deficient mice did not rescue cardiomyopathy [76]. Chemically modified RNA encoding *Tafazzin* was also able to rescue mitochondrial dysfunction in BTHS iPSC-CMs [19]. Adeno-associated viruse-9 (AAV9) mediated delivery of *Tafazzin* under the control of a myogenic-restricted desmin, a ubiquitously driven human cytomegalovirus immediate-early enhancer and promoter (CMV) or a native *Tafazzin* promoter each improved mitochondrial morphology, mitochondrial function, cardiac function and skeletal muscle performance in the *Tafazzin* knockdown mouse model [77]. In a separate study, AAV9-mediated delivery of *Tafazzin* under the control of the desmin promoter was able to normalize the proteomic profile of *Tafazzin*-deficient hearts [78]. AAV9-TAZ gene therapy was also able to restore mitochondrial morphology and function in BTHS patient fibroblasts [79]. AAV9-TAZ therapy administered to neonatal *Taz* KO mice was able to improve survival, reduce fibrosis, LV dilation and delay onset of cardiomyopathy when under the control of a *CMV* promoter but not a cardiomyocyte-restricted *cTNT* promoter, suggesting that the replacement of *Tafazzin* activity in skeletal muscle improves survival. It was also able to prevent the onset of cardiomyopathy and reverse established cardiomyopathy in *Tafazzin* myocardial conditional knockout mice when given at high doses [17].

## 7. Conclusions and Future Directions

*Tafazzin* is an essential regulator of mitochondrial structure and function in a variety of evolutionarily diverse organisms, through its ability to regulate the transacylation and maturation of cardiolipin in the inner mitochondrial membrane. Loss of *Tafazzin* function is manifest in striated muscle but also in other cells such as hematopoietic cells, spermatids and fibroblasts. Profound loss of function effects on development in zebrafish, mice and humans but not fruit flies indicate an important role in vertebrate development. The effects on mitochondrial metabolism are complex and substrate dependent. In BTHS, the effects on cardiac and skeletal muscle contractile function have been studied extensively, but the pathogenesis of neutropenia continues to be poorly understood. Gene therapy is promising in preclinical models. Future studies on the contribution of *Tafazzin* to early embryo, early cardiac development, hematopoietic development, male germ cell and isolated skeletal muscle function all await future conditional knockout mouse experiments. Elucidation of the contribution of *Tafazzin* isoforms to mitochondrial function await selective cDNA knock in rescue experiments of global and conditional knockout mice. Understanding of the myriad human variants and their contribution to BTHS await future knock in experiments in mice. Moreover, the crystal structure, the precise localization/s of *Tafazzin* within mitochondria, the scope of protein interactions it is engaged in, and its biological function are all still yet to be solved and/or determined [80]. Thus, further multidisciplinary approaches will be required to understand this myriad of intriguing unanswered *Tafazzin* and BTHS-associated questions.

## Figures and Tables

**Table 1:** Lists the species and their associated major phenotypes, as well as the type of genetic manipulation and whether it results in absent *Tafazzin*, reduced wildtype *Tafazzin* or presence of mutant *Tafazzin* protein.

Model Species	Genetic Manipulation	Major Phenotypes
*Saccharomyces cerevisiae* (yeast)	*Taz1∆* null mutation (no Taz)	temperature-sensitive growth, mitochondrial abnormalities, abnormal cardiolipin remodeling [8]
*Drosophila melanogaster* (fruit fly)	excision of upstream P element in *Taz* coding region (no Taz)	reduced locomotor activity, mitochondrial abnormalities, cardiolipin deficiency, defective spermatogenesis [9]
*Danio rerio* (zebrafish)	morpholino knockdown of *Taz* (reduced wildtype Taz)	dose-dependent embryonic lethality, growth retardation, abnormal heart formation and function [10]
*Mus musculus* (mouse)	CRISPR-generated *Taz* exon 3 knockout in immortalized C2C12 myoblast line (no Taz)	impaired myocyte differentiation, mitochondrial abnormalities, cardiolipin deficiency, increased mitochondrial ROS production [11]
	*Taz* short hairpin RNA knockdown (reduced wildtype Taz)	variable male embryonic lethality, developmental growth retardation, mitochondrial abnormalities, cardiolipin deficiency, abnormal heart formation, adult heart failure [12,13,14]
	high % *Taz^wt^/Taz^KO^* chimeras selected by coat color (reduced wildtype Taz)	male growth retardation, abnormal cardiolipin remodeling, defective spermatogenesis [15,16]
	*Taz* exons 5–10 loxP flanked global knockout (no Taz)	extensive male embryonic and neonatal lethality, growth retardation mitochondrial abnormalities, abnormal cardiolipin remodeling, poor adult cardiac function, cardiac and skeletal muscle defects [9,17]
	*Taz* exons 5–10 loxP flanked cardiomyocyte specfic knockout (no Taz in cardiac myocytes)	normal survival of mutant males, abnormal cardiolipin remodeling, mitochondrial abnormalities, reduced cardiac function, myocardial fibrosis and cardiomyocyte apoptosis [16,17]
*Homo sapiens* (human)	BTHS male patient skin fibroblasts (mutant Taz present)	abnormal cardiolipin remodeling, mitochondrial abnormalities [18]
	BTHS male patient induced pluripotent stem cells (iPSCs) (mutant Taz present)	iPSCs-cardiomyocytes exhibit abnormal cardiolipin remodeling, mitochondrial abnormalities, increased ROS production, sarcomere assembly and myocardial contraction abnormalities [19]

**Table 2:** Lists the most common male BTHS patient phenotypes and their possible in utero and perinatal developmental origins.

Common BTHS Patient Phenotypes *	Potential Developmental Origin/s of Phenotypes
male miscarriage and stillbirths	zebrafish and male mouse knockdown/knockout studies indicate that *Tafazzin* deficiency leads to profound cardiac developmental defects incompatible with survival
cardiolipin abnormalities and mitochondrial morphological and functional defects	defective CL remodeling results in abnormal embryonic mitochondrial morphogenesis, maturation, numbers, biogenesis and/or function with consequences for organ development
growth retardation/short stature	functioning mitochondria are essential for successful fetal development and intrauterine growth.
increased levels of 3-methylglutaconic acid in blood/urine	reduced mitochondrial energy production results in 3-methylglutaconic acid accumulation that can lead to metabotoxic effects in developing organs
neutropenia (absent to severe; persistent or cyclical)	reduced mitochondrial function affects the myeloid precursors leading to reduced production of mature neutrophils
dilated cardiomyopathy (often with left ventricular noncompaction) and/or endocardial fibroelastosis	reduced mitochondrial function leads to poor cardiac function, maturation and remodeling, with subsequent susceptibility to injury, manifest as ectopic lipid deposits, cardiac fibrosis, ventricular arrhythmia, prolonged QTc intervals, and ventricular dilation
skeletal myopathy	reduced mitochondrial function affects skeletal muscle maturation leading to smaller myocyte and muscle fiber sizes and reduced muscle strength

* Although most BTHS patients are male there are reports of affected TAZ female carriers [66,67,68].
